# Nursing Care Interpersonal Relationship Questionnaire: elaboration and validation ^1^

**DOI:** 10.1590/1518-8345.2128.2962

**Published:** 2018-01-08

**Authors:** José Wicto Pereira Borges, Thereza Maria Magalhães Moreira, Dalton Franscisco de Andrade

**Affiliations:** 2 PhD, Adjunct Professor, Universidade Federal do Piauí, Floriano, PI, Brazil.; 3 PhD, Adjunct Professor, Centro de Ciências da Saúde, Universidade Estadual do Ceará, Fortaleza, CE, Brazil.; 4 PhD, Full Professor, Departamento de Informática e Estatística, Universidade Federal de Santa Catarina, Florianópolis, SC, Brazil.

**Keywords:** Interpersonal Relation, Validation Studies, Nursing Care, Psychometrics, Dimensional Measurement Accuracy, Nursing Theory

## Abstract

**Objective:**

to elaborate an instrument for the measurement of the interpersonal relationship in nursing care through the Item Response Theory, and the validation thereof.

**Method:**

methodological study, which followed the three poles of psychometry: theoretical, empirical and analytical. The Nursing Care Interpersonal Relationship Questionnaire was developed in light of the Imogene King’s Interpersonal Conceptual Model and the psychometric properties were studied through the Item Response Theory in a sample of 950 patients attended in Primary, Secondary and Tertiary Health Care.

**Results:**

the final instrument consisted of 31 items, with Cronbach’s alpha of 0.90 and McDonald’s Omega of 0.92. The parameters of the Item Response Theory demonstrated high discrimination in 28 items, being developed a five-level interpretive scale. At the first level, the communication process begins, gaining a wealth of interaction. Subsequent levels demonstrate qualitatively the points of effectiveness of the interpersonal relationship with the involvement of behaviors related to the concepts of transaction and interaction, followed by the concept of role.

**Conclusion:**

the instrument was created and proved to be consistent to measure interpersonal relationship in nursing care, as it presented adequate reliability and validity parameters.

## Introduction

Interpersonal relationship in nursing care can be defined, based on a theory ^1^, as the interaction between two or more people who communicate, transfer values and energy from their roles in society. Such interaction is continuing, ranging from a low to high effectiveness ratio in nursing care. This subjective essence demands the search for support for its evaluation. 

In this sense, the measurements available in the literature refer to general interpersonal relationship and to instruments derived from psychology, such as: Relationship Inventory, Vanderbilt Psychoterapy Process Scale; Penn Alliance Scales; the Working Alliance Inventory; California Psychotherapy Alliance Scales^2^ ^-^ ^3^. In Brazil, there are the Scale for Emotional Contagion, Social Skills and Emotional Intelligence, Inventory of Empathy and the Davis’ Multidimensional Scale of Interpersonal Reactivity^4^ ^-^ ^5^. However, on the interpersonal relationship in nursing care, no instruments were found, which indicated lack of knowledge and lack of scales in this area. In addition, in the United States, in the nursing scope, the instrument found was the Interpersonal Communication Assessment Scale^6^, used to measure communication between undergraduate and graduate students and also validated for Portugal^7^. This scale is restricted to the communication process and has students as the target audience. 

Given the above, the instruments used to measure the interpersonal relationship do not relate to nursing, have different conceptual directions, mostly from psychology, and only deal with parts of this construct, such as communication and empathy^4^ ^,^ ^8^. In turn, the scarcity of instruments measuring interpersonal relationship in nursing care makes it difficult to evaluate specific elements of the nursing work that make the interpersonal relationship effective. 

Therefore, the measurement of interpersonal relationship in nursing care remains an open field for research. The development of an instrument in this area would not only identify the current stage of this interpersonal relationship in nursing care, but it would also provide parameters to improve it, favoring a humanitarian praxis, based on general health promotion, prevention of suffering and improvement of care by allowing a system to monitor the quality of interpersonal relationships. Thus, the objective of this study was to elaborate an instrument for the measurement of the interpersonal relationship in nursing care through the Item Response Theory (IRT), and the validation thereof.

## Method

It is a methodological study, with a quantitative approach, delineated from the theoretical, empirical and analytical poles of Psychometrics^9^ ^-^ ^10^. In the theoretical pole, the theoretical dimensionality was defined and the constitutive and operational definitions were established. The items were elaborated and content validation was carried out. 

The theoretical dimensionality was defined based on the concepts that make up the Interpersonal System of the Imogene King’s Interacting Open Systems Model, which proposes that interpersonal relationship is composed of five constitutive elements: interaction, communication, transaction, role and stress^1^. These elements were carefully analyzed and the constitutive definitions emerged from them.

After elucidating the constitutive definitions, the operational definitions and the items were elaborated, based on an integrative revision^11^ ^)^ and on six focus groups, considering the variety of nursing actions and their contexts, which occurred in the three levels of health care. The Primary Care groups took place in a Primary Health Care Unit (PHCU) that is run by the Ministry of Health’s standard programs and performs low complexity care, education and health promotion. The Secondary Care groups were carried out at the Integrated Center for Hypertension and Diabetes (ICHD), a reference unit for research and care on these diseases, where all its users go through a nursing consultation and health education sections with the nurse. The groups performed at the Walter Cantídio University Hospital (WCUH) represented the Tertiary Care. The WCUH is a reference center for high-complex care, human resources training and research development. All these public services are located in the city of Fortaleza-Ceará-Brazil. 

The inclusion criteria for the participants were: at the PHCU and at the ICHD, individuals aged> 18 years, who had been followed for at least one year in the service and who were waiting for the nursing consultation. In the WCUH, the inclusion criteria were individuals> 18 years of age, having been hospitalized for at least 24 hours in the wards. Those who did not communicate verbally and those who were in isolation for some contagious infectious disease that prevented interaction with the researcher were excluded.

The elaboration of the items followed the twelve criteria of psychometry (amplitude, balance, behavior, simplicity, clarity, relevance, precision, modality, typicity, objectivity, variety and credibility)^9^ ^-^ ^10^. The Nursing Care Interpersonal Relationship Questionnaire (NCIRQ) was elaborated with 44 items and a four-point adjectival scale (never, sometimes, most of the time and always). 

Afterwards, the content validation of the NCIRQ was performed, with content and semantic analysis. The content analysis was performed by nine nurses that are experts in interpersonal relationship. These were five academics and four clinicians with clinical experience, research and publications on the subject, from four Brazilian states. Initially, the Coordination for Improvement of Higher Education Personnel (CAPES in Portuguese) database was searched for people who studied “interpersonal relationship in nursing” and then other experts were identified and contacted. To determine the level of agreement, the Content Validity Index (CVI) was ≥0.78^12^. 

In order to perform the semantic analysis, the NCIRQ was applied in a pilot test to 66 people in the same locations of the focus groups and considering the same inclusion and exclusion criteria, with 28 people from primary care, 23 from secondary care and 15 from tertiary care. These people were distinct from focus group participants. The pilot sample was constituted considering the minimum parameter of 5% of the sample of the empirical phase. The difficulties in understanding the words and expressions present in the items were observed, participants were asked about the need for adjustments, and the adequacy of the response categories of each item was reviewed. 

In the empirical pole, the planning and application of the NCIRQ was carried out in order to evaluate its psychometric properties through the TRI. This stage was carried out in six PHCUs that were randomly selected, in the ICHD and in the WCUH, considering the same inclusion and exclusion criteria delineated for the focus groups. 

Participants in studies for the development of instruments via TRI should have a sample size enough to preserve heterogeneity and achieve respondents covering the entire latent trait, but there is no consensus on the ideal number. Simulations for the decision of sample size in TRI concluded that 500 subjects in relation to 40,000 bring results very close to those estimated in larger samples^13^. In this sense, an average of 20 respondents were agreed for each of the 44 items, totaling 880 people, plus 10% to repair losses, thus reaching 968 people. Considering the application of the instrument at the three health care levels, the NCIRQ was applied to 950 patients, 319 of which were in the PHCU, 335 in the ICHD and 296 in the WCUH. There were 18 participants lost due to incompleteness in the answers to the items, but this was within the expected range.

The NCIRQ was applied by six nurses with research experience and previously trained. The training was carried out in a private room, in which the objectives of the research were presented, as well as the NCIRQ. On another day, the researchers were accompanied by the research coordinator at one of the data collection sites, where they observed and applied the NCIRQ. In another day, again in a private room, a meeting was carried out to clear the doubts. Data collection occurred during the period from May to September 2015.

In the analytical pole, the steps for the analysis of the psychometric properties of the NCIRQ were conducted. In the reliability analysis, internal consistency was verified by the Cronbach’s and McDonald’s Omega coefficients, whose reference values for these measures were: <0.6 - low; between 06 and 0.7 - moderate; and between 0.7 and 0.9 - high reliability^14^. 

The McDonald’s Omega coefficient was used to verify the maintenance of the Tau-equivalence principle. This coefficient is a better measure of reliability when the Tau-equivalence principle is violated, that is, when the items do not show similar values in the coefficient matrix; its reading is similar to that of Crombach’s Alpha and should be performed in comparison, since a low Alpha value followed by a high Omega value indicates such a violation, the latter being the coefficient that best demonstrates reliability^14^. 

The dimensionality study was done on the polyclonal correlation matrix and the main components were analyzed, with oblimim rotation and parallel analysis^15^. These analyzes were performed using the statistical packages “Rcmdr” ^(^ ^16^ and “psych” of R^17^. In order to establish the presence of a dominant dimension in the NCIRQ, the convention was adopted that a variance explained by the first factor greater than 20% indicates essential unidimensionality^18^.

In the estimation of parameters, the one-dimensional Gradual Response Model of TRI was applied and it was performed in the Multilog software to observe the estimates of the standard errors of the parameters, subsidizing the decisions of exclusion of items, and confirmed by using the package “mirt” of R^19^. Regarding the interpretation of parameter *a* (item discrimination), values above 0.6 are acceptable, and the higher the value of *a*, the greater the discrimination power of the item; for parameter *b* (difficulty/positioning), the values are acceptable in the range of -5 to +5 ^20^. 

The scale construction was performed based on the anchor levels of the categories of items with good discrimination (a> 0.6). The anchor levels are points on the scale selected to be interpreted and the anchor items are those selected for each of the anchor levels^21^. For an item to be considered anchor at a given level of the scale, it is expected to be positively answered by at least 65% of the respondents and by a proportion less than 50% of those with the immediately lower level. The difference between the proportion of these two levels should be at least 30%^20^. Because it was difficult to meet all conditions, item categories were positioned at the 60% response rate (near-anchor levels).

After estimating the parameters, the Test Information Function (TIF) was elaborated. A skill scale was established, defining a source and a measure unit for the scale definition. Initially, the parameter values of the items (*a, b*) and of the scores were estimated in the same metric in the scale with mean 0 (zero) and standard deviation 1 (one). Then, these values were transformed using mean 50 and standard deviation 5, scale (50, 5), to improve understanding of the results. 

The present study was approved by the Research Ethics Committees of the State University of Ceará (opinion No. 984723) and of the WCUH (opinion No. 1048399).

## Results

The analysis of the Interpersonal System of the Interacting Open Systems Model revealed the theoretical dimensionality composed by the constitutive definitions of the concepts interaction, communication, transaction, role and stress. Based on these concepts, the Nursing Care Interpersonal Relationship Questionnaire (NCIRQ) was composed of 44 items, nine from the interaction concept, eleven from communication, nine from transaction, eight from role and seven on stress. Content validation revealed that all items had excellent CVI (≥0.78) and were comprehensible, representing a good theoretical delineation.

**Figure 1:**
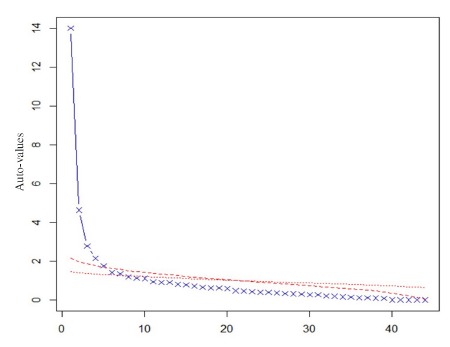
Main Components Analysis scree plot with parallel analysis in the polycritical correlation matrix of the NCIRQ*. Fortaleza, CE, Brazil, 2015

After its application, the NCIRQ, composed of 44 items, had Cronbach’s alpha of 0.86 and McDonald’s Omega of 0.90. The study on dimensionality showed a dominant dimension, explaining 31.5% of the variance of the items responses (Figure 1), indicating essential unidimensionality, a necessary condition to build a one-dimensional scale based on TRI.

* Nursing care interpersonal relationship questionnaire

After the essential unidimensionality was detected, the item parameters were estimated. Five calibrations were carried out until the definition of the final model. The first attempt was made with all NCIRQ items and did not achieve algorithm convergence. The results showed a grouping of items with burst standard errors (items 33 to item 41 representing the stress concept) that could be preventing convergence and hindering the accuracy of the parameters of items 13, 27, 32 and 42, with high errors in the estimates. 

The second calibration attempt was performed with 35 items. Items 33 to 41, representing the stress concept, presented in a different dimension of the latent trait and had to be withdrawn. The 35-item model presented convergence, confirming that items 33 to 41 were preventing calibration. However, items 13, 27 and 43 presented low rates of discrimination with high standard errors, indicating that they were not part of the latent trait and were eliminated. 

The third attempt was made with 32 items, and convergence was achieved. However, item 32 presented a high standard error associated with the difficulty parameter of the item, being removed from the NCIRQ. The fourth calibration attempt was performed with 31 items. In this attempt, the parameters of all the items presented low standard errors, indicating good modeling by TRI. A fifth calibration was necessary with the 31 items, because in the anchoring process of items 02, 10, 12, 19, 22, 24, 28, 30 and 31 there was level overlap due to the proximity of parameters *b*. They were re-categorized in three response categories. Finally, the Mirt package of software R was used to confirm the model with 31 items, obtaining convergence after 40 cycles. 

**Figure 2:**
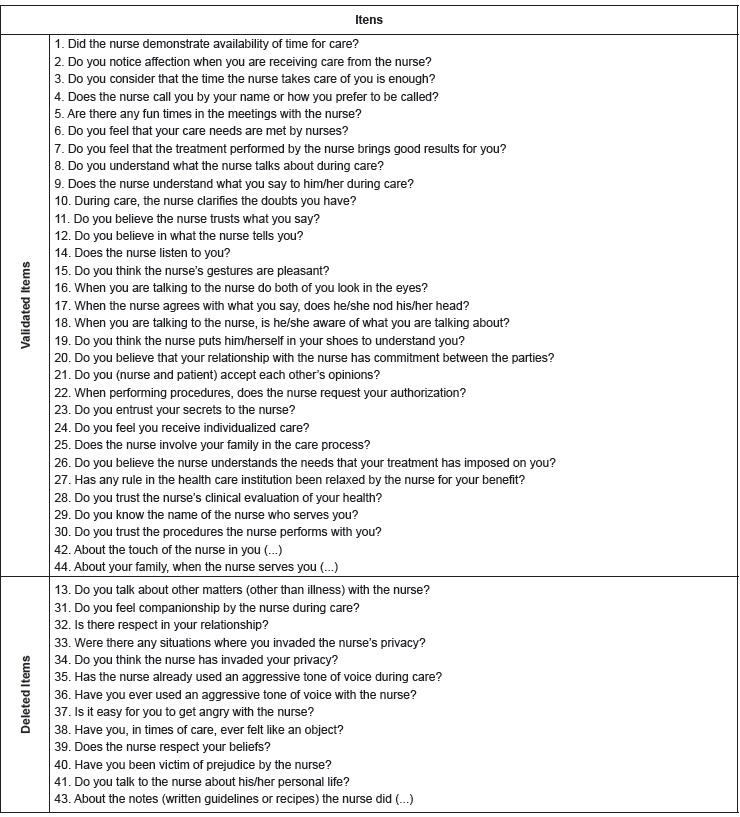
Items of the Nursing Care Interpersonal Relationship Questionnaire. Fortaleza, CE, Brazil, 2015

After the 13 items were removed, the reliability was again tested and an improvement was observed in Cronbach’s alpha, which increased from 0.86 to 0.90 and the McDonald’s Omega increased from 0.90 to 0.92, demonstrating that the violation of the Tau equivalence was of little magnitude, since the withdrawal of items increased only 0.2 in the latter index. Figure 2 shows the NCIRQ items, indicating items 13, 31 to 41 and 43 that were eliminated in the calibration process.

The estimates of the parameters of the items are shown in Table 1, which presents the 31 items of the final instrument and the parameters *a*, discrimination, and *b2, b3* and *b4*, difficulty, for the category sometimes, most of the times and always on the adjectival scale.

**Table 1:** Estimation of the parameters of the NCIRQ* items in a sample composed of Primary, Secondary and Tertiary Care patients. Fortaleza, CE, Brazil, 2015

**Items**	**a†**	**b2‡**	**b3§**	**b4||**
**1. Did the nurse demonstrate availability of time for care?**	**1.62**	**-2.68**	**-1.58**	**-1.33**
**2. Do you notice affection when you are receiving care (...)**	**2.00**	**-2.66**	**-1.22**	
**3. Do you consider that the time the nurse takes care (...)**	**1.68**	**-2.35**	**-1.48**	**-1.26**
**4. Does the nurse call you by your name (...)**	**1.21**	**-2.75**	**-2.18**	**-1.97**
**5. Are there any fun times in the meetings with the nurse?**	**1.02**	**-1.01**	**0.43**	**0.82**
**6. Do you feel that your care needs are met by nurses?**	**2.59**	**-2.18**	**-1.24**	**-1.03**
**7. Do you feel that the treatment performed by the nurse (...)**	**2.15**	**-2.37**	**-1.43**	**-1.19**
**8. Do you understand what the nurse talks about during care?**	**1.09**	**-5.23**	**-2.31**	**-1.94**
**9. Does the nurse understand what you say (...)**	**1.33**	**-3.87**	**-2.69**	**-2.12**
**10. During care, the nurse clarifies the doubts you have?**	**1.35**	**-2.15**	**-1.37**	
**11. Do you believe the nurse trusts what you say?**	**1.23**	**-3.63**	**-2.06**	**-1.65**
**12. Do you believe in what the nurse tells you?**	**2.08**	**-2.49**	**-1.53**	
**14. Does the nurse listen to you?**	**1.74**	**-3.10**	**-1.90**	**-1.63**
**15. Do you think the nurse’s gestures are pleasant?**	**2.11**	**-2.61**	**-1.37**	**-1.15**
**16. When you are talking to the nurse do both of you (...)**	**1.55**	**-2.40**	**-0.86**	**-0.69**
**17. When the nurse agrees with what you say (...)**	**1.19**	**-2.38**	**-1.48**	**-1.27**
**18. When you are talking to the nurse (...)**	**2.36**	**-2.69**	**-1.38**	**-1.21**
**19. Do you think the nurse puts him/herself in your shoes (...)**	**1.42**	**-0.81**	**-0.24**	
**20. Do you believe that your relationship with the nurse (...)**	**1.86**	**-2.18**	**-1.25**	**-1.00**
**21. Do you (nurse and patient) accept each other’s opinions?**	**1.34**	**-2.68**	**-1.19**	**-0.88**
**22. When performing procedures, does the nurse request (...)**	**0.93**	**-0.54**	**-0.29**	
**23. Do you entrust your secrets to the nurse?**	**0.56**	**2.29**	**3.72**	**3.85**
**24. Do you feel you receive individualized care?**	**0.91**	**-1.46**	**-0.99**	
**25. Does the nurse involve your family in the care process?**	**0.64**	**-0.22**	**1.30**	**1.66**
**26. Do you believe the nurse understands the needs (...)**	**1.71**	**-2.08**	**-1.40**	**-1.13**
**28. Do you trust the nurse’s clinical evaluation of your health?**	**1.96**	**-2.34**	**-1.65**	
**29. Do you know the name of the nurse who serves you?**	**0.37**	**-0.54**	**1.43**	**1.96**
**30. Do you trust the procedures the nurse performs with you?**	**2.28**	**-2.34**	**-1.65**	
**31. Do you feel companionship by the nurse during care?**	**1.62**	**-1.52**	**-0.76**	
**42. About the touch of the nurse in you:**	**0.76**	**-3.10**	**-2.20**	
**44. About your family, when the nurse serves you:**	**0.51**	**-0.47**	**2.17**	**3.68**

*Nursing Care Interpersonal Relationship Questionnaire; †Item discrimination parameter; ‡Difficulty parameter of category 2 (sometimes); §Difficulty parameter of category 3 (most of the time); || Difficulty parameter of category 4 (always).

The items that best discriminated the patients regarding the effectiveness of the interpersonal relationship with the nurse were 06, 07, 18 and 30, with higher discrimination parameter, *a*. It was observed that items 23, 29 and 44 were below the criterion adopted because of the low discrimination. However, considering that these items did not disturb the calibration, they were kept in the instrument and excluded from the scale interpretation.

Regarding the difficulty of the item, its measure is given by parameter *b*, which indicates the position in the scale in which the item has more information. The higher the *b*, the greater the difficulty of this item. Thus, when reaching the items with greater value of *b*, the patient will have a more effective interpersonal relationship in the nursing care. Considering the items with discrimination above the reference value adopted, only item 25 presented a positive parameter *b*, which shows that NCIRQ items are easy for all respondents, a fact consistent with the latent trait studied, in which the behaviors evaluated integrate the daily routine of nurses and patients in the care process. 

**Figure 3:**
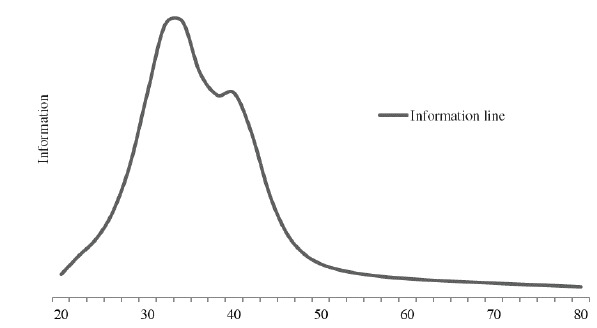
Test Information Function. Fortaleza, CE, Brazil, 2015.

Figure 3 shows the Test Information Function (TIF) on the transformed scale (50.5), which shows that the NCIRQ has higher information (higher curve) in the range of 25 to 45 points. This means that it is more appropriate to measure the level of interpersonal relationship in nursing care in patients who are in this range. It demonstrates, therefore, that the instrument is more indicated to measure the low effectiveness in the interpersonal relation.

From an interpretative point of view, each item, along with its response categories, representing the theoretical concepts, carry information for the interpretation of the latent trait. Thus, the construction of the scale interpretation is based on 62 indicators of interpersonal relationship in nursing care. Thus, the interaction concept contributed with 19 indicators, the communication concept with 20 indicators, the transaction concept with 18 indicators, and the role concept with 5 indicators. The stress concept did not contribute with indicators for the scale interpretation. 

**Figure 4:**
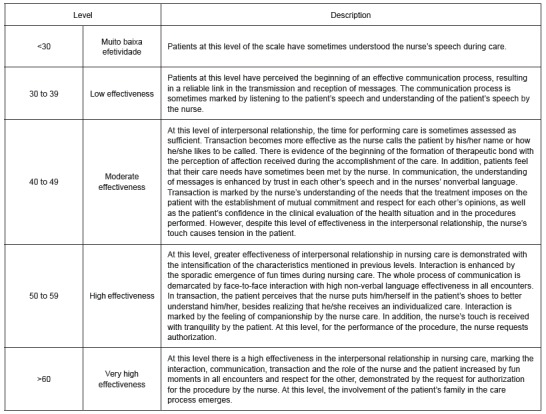
Nursing care interpersonal relationship scale. Fortaleza, CE, Brazil, 2015

Figure 4 shows the scale of interpersonal relationship in nursing care. Its first level is demarcated by understanding of the nurse’s speech during care as the initial link to care. At the next level (30 to 39 points), communication becomes a two-way street, establishing the communicative process. Then (40 and 49 points), the transaction gains body from the recognition of the patient’s identity by the nurse and interaction with the recognition of respect in care. At level 50 to 59 there is a deepening of the interpersonal relationship in care when the perception of an individualized care emerges in transaction and the feeling of companionship in interaction. Above 60 points, the interpersonal relationship goes beyond the tenuous threshold of tension of a discourse, essentially focused on the patient’s health situation, with the reporting of fun moments and family involvement, thus marking the upper level of the scale.

## Discussion

The elaboration of the NCIRQ was done in order to allow theoretical deepening and its empirical correspondence, since it involved the careful analysis of King’s model^1^ with an examination of the literature and the accomplishment of exploratory focus groups, enabling an association between the abstract concepts of the theory and measurable indicators, bringing contribution of information and culture involved in nursing care to the NCIRQ^22^.

On content validation, content analysis involving a group of experts is a consolidated technique in validation studies of nursing phenomena^23^. The selection of specialists from different regions allows adapting the instrument built for the country, considering the cultural diversity, which in studies of this type cannot be ignored^24^. The semantic analysis allowed evaluating some aspects of the measurement process that could affect the data collection, such as the format of the answers and the individual items^25^. This stage was important for the adequacy of the pilot instrument to the application in the three levels of health care. 

Regarding the analysis of psychometric properties, the NCIRQ demonstrated adequate internal consistency by Cronbach’s alpha and McDonald’s Omega. The comparison between these two coefficients assesses the bias by using either measure^14^. In this study, very close and high values indicate good reliability by the two indicators. On dimensionality, the NCIRQ has shown to have a dominant dimension. This is a relevant step in the research in order to identify suitable models for the understanding of the studied phenomenon^9^ ^-^ ^10^. 

Regarding the parameters of the items, there was a good performance referring to the concept of communication, transaction and interaction, since they presented a better power to discriminate individuals with more effective nursing care interpersonal relationship. 

But the items in the role concept that dealt with trusting secrets, knowing the nurse’s name, and the family’s approach were the ones that least differentiated patients in the latent trait and contributed with less psychometric information on the instrument. The amount of information of the item provides indication of the accuracy of the measurement associated with each level of the scale^9^ ^-^ ^10^ ^,^ ^21^. An alternative to this result would be to add well-formulated items of the role concept to increase information and improve the measurement of individuals in aspects of this concept. It is also necessary to carry out studies on stress to clarify their real involvement as a dimension of the latent trait, since the items of this concept were eliminated from the NCIRQ. 

On parameter *b*, the NCIRQ results show almost all the difficulty parameters (*b*) as negative. It is supposed that, since the behaviors contained in the items are inherent to the human interactions and to the daily life of the people receiving nursing care, and thus practiced by those involved in the process without great efforts, it has culminated with negative parameters *b,* in its large majority. A similar result was found in a study involving the construct “comfort of relatives of critically ill people”^25^. 

In the case of TIF, a reliability measure of TRI^26^, the NCIRQ presented better reliability to measure interpersonal relationship in low to medium levels of effectiveness. This does not detract from its relevance, since low-effectiveness interpersonal relationships in care generate greater concern for nurses than those with high effectiveness.

According to the constructed scale, the evolution of the effectiveness of interpersonal relationship in nursing care in five levels was evident. At the first level, the communication process begins. The following levels demonstrate qualitatively the points of effectiveness of the interpersonal relationship with the involvement of behaviors related to the concepts of transaction and interaction, followed by role. This interpretation of levels is a characteristic of the TRI models, which enables the creation of a plan of care for the patient, according to their individual score^21^.

It is worth mentioning that interpersonal relationship is a basic tool of care in nursing and, therefore, is a fundamental skill for the performance of the entire professional activity. Seeing the interpersonal relation from the perspective of the production of a measurement technology is to give subsidy to the profession as a way of evaluating its daily behavior, allowing space so that the knowledge of situations generates improvement in the interaction between nurse and patient.

The components of interpersonal relationship become paramount in the development of care with a view to its humanization, pointing to the need for constant training of nurses involved in the care process, not only in technical procedures, but especially in their better qualification for the development of safe interpersonal relationships, learned as professional care tools^27^. In this sense, the measurement of interpersonal relationship can be used both to evaluate competence and to strengthen these skills in groups or individuals, as these can be improved with instruction and modified over time^28^ ^-^ ^29^. In addition, this instrument can be used to improve understanding of the communication process.

A study that developed an instrument to measure communication was shown to be important in different situations and to provide guidelines for individual or group intervention with the purpose of improving relationships and well-being in the context of health services, as well as to reflect on the theme in an educational manner^29^. 

Regarding limitations, not all concepts of theory remained represented in the final instrument. In addition, it is necessary to formulate items that anchor at the upper levels of the scale, improving the measurement at these levels. 

## Conclusion

The NCIRQ was built on the framework of the Interpersonal System of the Interacting Open Systems Model, resulting in an instrument with 31 items and a five-level interpretive scale. It demonstrated content validity and showed high internal consistency. The instrument was analyzed in its dimensionality and via TRI, in which its validity was demonstrated. The visualization of the parameters of the items and their individual contributions in the measurement of the latent trait allowed the construction of a scale with an interpretative model that shows essentially how effective the interpersonal relation in the nursing care is. The construction of an interpretation for each level of the scale is configured as filling the gap in the health behavior measurement studies, going beyond the answers commonly provided by such instruments. As the instrument was validated via TRI and resulted in an interpretive scale, the results of its application can be comparable because they have in their structure of analysis the same metric for measuring the latent trait.

The use of the NCIRQ will allow new interpretive horizons, both in clinical practice and in research on interpersonal relationship in nursing. Its results can be used as adjuncts in the evaluation of the quality of care, as well as in the redirection of daily practices that promote greater effectiveness of the interpersonal relation in nursing in the health services. It is hoped that the use of the NCIRQ may support actions that may contribute to the development of strategies that facilitate more effective interpersonal relationships in nursing care.
